# Genetic variation among elite inbred lines suggests potential to breed for BNI-capacity in maize

**DOI:** 10.1038/s41598-023-39720-3

**Published:** 2023-08-17

**Authors:** César D. Petroli, Guntur V. Subbarao, Juan A. Burgueño, Tadashi Yoshihashi, Huihui Li, Jorge Franco Duran, Kevin V. Pixley

**Affiliations:** 1 International Maize and Wheat Improvement Center (CIMMYT), Carretera México-Veracruz, Km. 45, El Batán, Texcoco, C.P. 56237 Mexico; 2 Japan International Research Center for Agricultural Science, 1-1 Ohwashi, Tsukuba, Ibaraki 305-8686 Japan; 3 Institute of Crop Sciences, Chinese Academy of Agricultural Sciences (CAAS), No 12 Zhongguancun South Street, Beijing, 10081 China; 4 Departamento de Biometría y Estadística, Facultad de Agronomía, Universidad de la República, Ruta 3, Km 363, C.P. 60000 Paysandú, Uruguay

**Keywords:** Genetics, Physiology, Plant sciences, Systems biology

## Abstract

Biological nitrification inhibition (BNI) is a plant function where root systems release antibiotic compounds (BNIs) specifically aimed at suppressing nitrifiers to limit soil-nitrate formation in the root zone. Little is known about BNI-activity in maize (*Zea mays* L.), the most important food, feed, and energy crop. Two categories of BNIs are released from maize roots; hydrophobic and hydrophilic BNIs, that determine BNI-capacity in root systems. Zeanone is a recently discovered hydrophobic compound with BNI-activity, released from maize roots. The objectives of this study were to understand/quantify the relationship between zeanone activity and hydrophobic BNI-capacity. We assessed genetic variability among 250 CIMMYT maize lines (CMLs) characterized for hydrophobic BNI-capacity and zeanone activity, towards developing genetic markers linked to this trait in maize. CMLs with high BNI-capacity and ability to release zeanone from roots were identified. GWAS was performed using 27,085 SNPs (with unique positions on the B73v.4 reference genome, and false discovery rate = 10), and phenotypic information for BNI-capacity and zeanone production from root systems. Eighteen significant markers were identified; three associated with specific BNI-activity (SBNI), four with BNI-activity per plant (BNIPP), another ten were common between SBNI and BNIPP, and one with zeanone release. Further, 30 annotated genes were associated with the significant SNPs; most of these genes are involved in pathways of “biological process”, and one (AMT5) in ammonium regulation in maize roots. Although the inbred lines in this study were not developed for BNI-traits, the identification of markers associated with BNI-capacity suggests the possibility of using these genomic tools in marker-assisted selection to improve hydrophobic BNI-capacity in maize.

## Introduction

Massive application of nitrogen (N) fertilizers to agricultural crops, including maize, has economic and ecological implications ranging from its important contribution to food production and the “green revolution”^1^, to negative impacts on water pollution, depletion of soil-fertility, and greenhouse gas emissions^2^. Reducing N losses from N fertilizers will improve N uptake and N use efficiency, and can improve crop productivity^3,4^. Biological nitrification inhibition (BNI) is one approach to reduce soil N losses and is a plant-based natural process that can reduce N fertilizer demand while sustaining agricultural systems^5,6^. Several studies have reported BNI-activity in forage grasses^7–12^, legumes^13–15^, and cereal crops^8,16–20^, suggesting the possibility of selection and genetic improvement for this trait.

Plant roots release two classes of BNI-compounds, hydrophobic and hydrophilic; this categorization is based on their relative solubility in and affinity for water. Hydrophobic BNI-compounds are mostly confined to and active in the rhizosphere, whereas hydrophilic BNI compounds’ impact may extend beyond the rhizosphere^3^. Together, i.e., hydrophobic and hydrophilic BNI-compounds determine the BNI-capacity (i.e. nitrification inhibitory capacity) of root systems. The relative contributions of hydrophobic and hydrophilic BNI-compounds to the BNI-capacity of root systems may vary among plant species. For example, hydrophobic BNIs contribute about 60% of BNI-capacity in sorghum, whereas hydrophilic BNIs contribute nearly 90% of BNI-capacity in wheat^3^. Root systems of a wild wheat (*Leymus raceamosus* (Lam.) Tzvelev) have nearly 20-times higher BNI-capacity (i.e. ability to release BNI-activity from roots) compared to most cultivated wheats (*Triticum aestivum* L.); most of this inhibitory effect comes from hydrophilic-BNIs^16^. Recently, the chromosome region controlling production of BNIs from roots in *Leymus raceamosus* was identified and successfully transferred to elite wheat cultivars, without collateral effects on the elite agronomic features^21^. In rice (Oryza sativa), high BNI-activity was reported and the responsible compound was identified^17,20,22^. Recent reports suggest that in maize, hydrophobic BNI-activity released from root systems accounts for 30–40% of BNI-capacity of root systems; a major portion of this inhibitory effect is attributed to a novel compound, named as ‘zeanone’^23^. This compound was recognized as a 1,4-naphothoquinone with a redox-active bicyclic structure, similar to the activities in the 1,4-quinone (p-benzoquinone) structure of sorgoleone discovered in sorghum^18^. Sorgoleone is reported to block both ammonia monooxygenase (AMO) and hydroxylamine oxidoreductase (HAO) pathways^18^, the two enzymatic pathways that play critical roles in the oxidation of NH_4_^+^ to NO_2_^−^ in *Nitrosomonas* spp.^24^.

Identification and characterization of genetic diversity for traits of interest, such as BNI-capacity, is crucial for breeding programs and genetic improvement efforts. Molecular marker methods, including genome-wide association studies (GWAS)^25–33^ and mapping of quantitative trait loci (QTL)^34–40^ have enabled applications such as marker assisted selection (MAS) and genomic selection (GS), which are widely used to accelerate and increase the accuracy of maize improvement^41–45^. Recently, maize inbred lines developed by the International Maize and Wheat Improvement Center (CIMMYT) were characterized using molecular markers, and GWAS was applied to identify genomic regions associated with resistance to different diseases^46–50^, drought and heat stress tolerance^51,52^, and adaptation to diverse N environments^53^. Several genes were identified as linked with biological processes related to nitrogen use efficiency (NUE)^53–55^.

The objectives of the present study were to:

1. Assess the genetic variation among 250 CIMMYT maize lines (CMLs) for hydrophobic BNI-capacity (i.e. hydrophobic BN-compounds released from roots). We studied only hydrophobic BNI-capacity because phenotyping hydrophilic BNI-activity is prohibitively cumbersome (can phenotype only 10–15 lines per year), and because hydrophobic BNI accounts for nearly half of total BNI-capacity in maize.
2. Assess genetic variation for ‘zeanone’ (the major BNI-compound isolated/identified from root DCM wash, and responsible for a major portion of hydrophobic BNI-activity) release and its association with hydrophobic BNI-activity.
3. Identify molecular markers and potential candidate genes associated with hydrophobic BNI-capacity and/or zeanone release from maize root systems, paving the way for molecular marker-based strategies to allow the effective development of germplasm able to reduce nitrifier activity and soil-nitrate formation in maize production systems.

## Results

### Genetic variation for hydrophobic-BNI-capacity of CMLs

**Figure 1:**
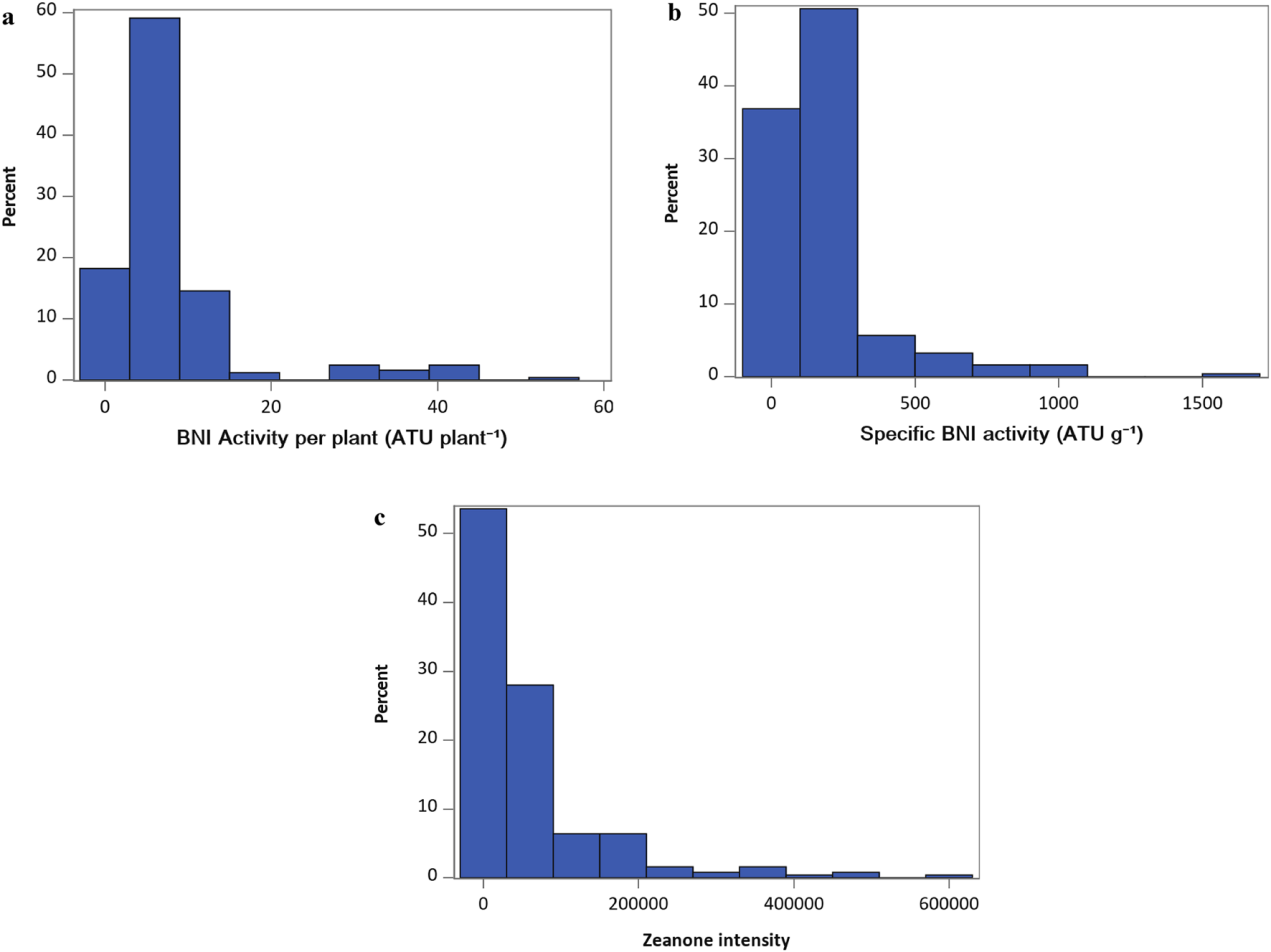
Histogram with CML mean of (**a**) BNI-activity per plant, (**b**) specific BNI-activity and (**c**) Zeanone intensity per plant. In all cases a clear skewed distribution is shown, with a small portion of the samples coming under highest BNI-capacity.

There was large phenotypic variation for hydrophobic BNI-activity among root systems of CMLs. The data for BNI per plant (BNIPP), specific BNI (SBNI) (BNI activity g^−1^ root dwt) and zeanone activity (zeanone levels in root exudate samples) had skewed distributions, with large variation; CVs larger than 100%. BNIPP, SBNI and zeanone had means of 8.10 ATU plant^−1^, 184.1 ATU g^−1^ and 59,458 zeanone intensity, respectively. The median was smaller than the mean for each trait, highlighting the skewed distribution of the data (Table 1). Most of the CMLs (90%) had BNIPP less than 12.5 ATU plant^−1^, but 10% had larger values, with a maximum of 54.5 ATU plant^−1^ (Fig. 1); the median of 5.2 ATU plant^−1^ is higher than observed for wheat (G.V. Subbarao, JIRCAS, unpublished results). Similarly, 90% of CMLs had SBNI between 0 and 344.5 ATU g^−1^, while the remaining 10% had values between 364.8 and 1604.4 ATU g^−1^. Zeanone intensity ranged from 579 to 624,377, and 90% of the lines had values below 164,354. Broad sense heritabilities exceeded 64% for all traits, and were close to 90% for SBNI and BNIPP, indicating strong repeatability for these data. Zeanone intensity per plant did not explain much of the variation (among 250 CMLs) for total hydrophobic BNI-activity, but BNIPP and SBNI were significantly, albeit weakly associated with this trait, R^2^ 7.64% and 8.36% (*P* < 0.001), respectively (see Supplementary Fig. S1 online). However, when we restrict this analysis to 50 CMLs (second batch), the relationship between total BNI-activity in the sample to zeanone levels improved (R^2^ 63%), indicating the possibility of existence of zeanone isomers among maize germplasm (see Supplementary Fig. S2 online).

**Table 1:** Descriptive statistics of phenotypic data obtained for hydrophobic BNI-activity per plant (BNIPP), specific BNI-activity (SBNI) and zeanone intensity per plant.

	BNIPP	SBNI	Zeanone
N	488	488	500
Mean	8.09	184.14	59,458
Median	6.23	122.68	22,211
Std Deviation	8.92	208.48	95,531
CV (%)	110.20	113.20	160.70
Maximum	56.35	1694	788,584
Minimum	0.00	0.00	21.39
Variance components*			
Genotypic variance	0.7159	0.7762	1.4499
Residual variance	0.1394	0.1494	1.6018
Heritability (%)	91.13	91.22	64.42

Variance components and heritability obtained by the mixed linear model.

N: Number of observations, 250 lines × 2 replicates.

Traits units: ATU plant^−1^ (BNIPP and SBNI); zeanone intensity.

*: values in ln-scale.

### GWAS analysis

As expected for inbred lines, molecular markers confirmed low frequencies of heterozygous loci and minor alleles, and little polymorphic information content for the CMLs, with averages of 0.02, 0.14 and 0.16, respectively (see Supplementary Fig. S3 online).

**Figure 2:**
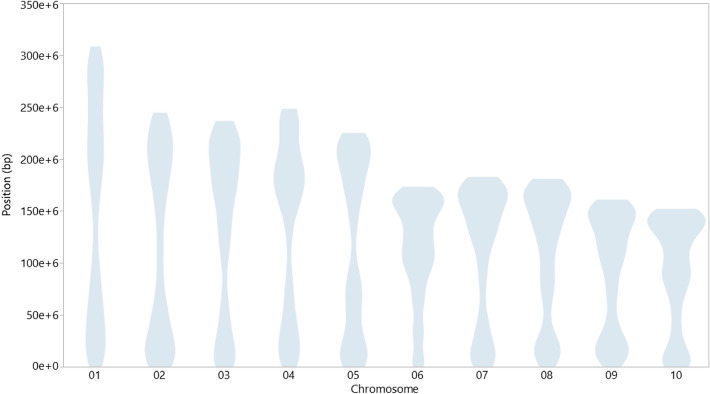
Violin plot showing SNP distribution based on physical position (bp) in each chromosome. There is a homogeneous distribution of the markers on the reference genome (B73 RefGen v.4).

We used the first two dimensions of a multidimensional scaling analysis on the CML MRD matrix to study the genetic diversity of the CMLs set and determine whether population structure required statistical correction. There was not a substantial genetic structure among the lines. Because of a continuum gradient in the distribution of the evaluated inbred lines (see Supplementary Fig. S4 online), we decided to include the first principal components dimensions in the GWAS analysis. Different number of components were tested (0, 2, 3, 5, and 10), and results were compared by the Bayesian Information Criterion (BIC) (see Supplementary Table S1 online). The SNPs positioned on the maize reference genome (B73 v.4) were homogenously distributed across all chromosomes, providing a good representation across the genome, and enabling the identification of genomic regions responsible for the expression of the evaluated traits (Fig. 2). An average of 2313 markers were found on each chromosome, with the largest number of SNPs (3688) on chromosome 1, and the smallest (1555) on chromosome 10 (see Supplementary Fig. S5 online).

**Figure 3:**
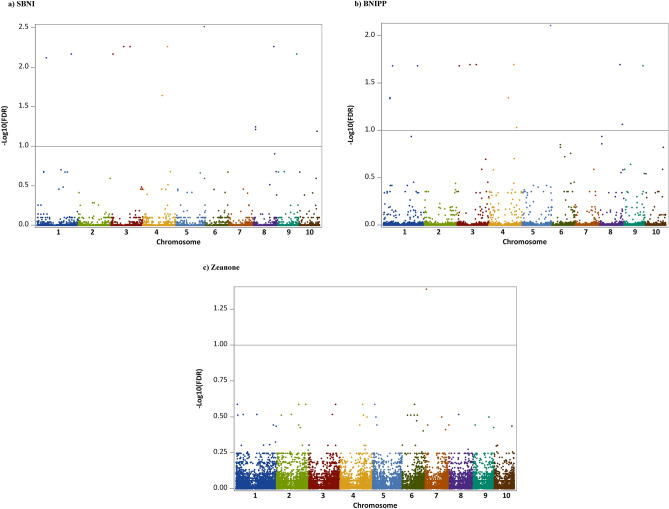
Manhattan Plot showing significant markers associated with: (**a**) Specific BNI-activity (SBNI); (**b**) BNI-activity Per Plant (BNIPP); and (**c**) Zeanone intensity. The horizontal scale shows the physical position of each SNP on the reference genome B73 (RefGen v.4). Significant markers (18) are distributed across all chromosomes.

GWAS analysis, using a false discovery rate of 0.10, revealed 18 significant markers associated with hydrophobic BNI-activity (Fig. 3). Three SNPs were significant exclusively for specific BNI (SBNI), four for BNI per plant (BNIPP), and only one for zeanone intensity per plant, while, ten SNPs were significant for both, SBNI-activity and BNIPP-activity (Supplementary Table S2 online). Significant SNP-trait associations were found on most of the chromosomes, with exceptions in chromosomes 2 and 6, ranging from 4 SNPs on chromosomes 1 and 8 to one each on chromosomes 5, 7, 9 and 10. The SNP with largest effect (100056639|F|0–15:A > G-15:A > G) had R^2^ = 0.15 for SBNI and R^2^ = 0.14 for BNIPP, and was located on chromosome 3. For zeanone intensity per plant, the significant marker 24028259|F|0–13:A > G-13:A > G had R2 = 0.12, and was positioned on chromosome 7.

Markers associated with SBNI were located on chromosomes 1 (2 SNPs), 3 (3), 4 (2), 5 (1), 8 (3), 9 (1), and 10 (1); while those significant for BNIPP were located on chromosomes 1 (4 SNPs), 3 (3), 4 (3), 5 (1), 8 (2), and 9 (1). Moreover, ten SNPs had pleiotropic effects for SBNI and BNIPP, indicating potential to select simultaneously for both traits in a breeding program.

### Linkage disequilibrium and putative genes

No linkage disequilibrium (LD) decay distance between GWAS-significant markers was shorter than 1Mbp (see Supplementary Fig. S6 online). This was not surprising because the germplasm was produced independently of each other, under strong selective procedures for traits other than those evaluated here.

**Figure 4:**
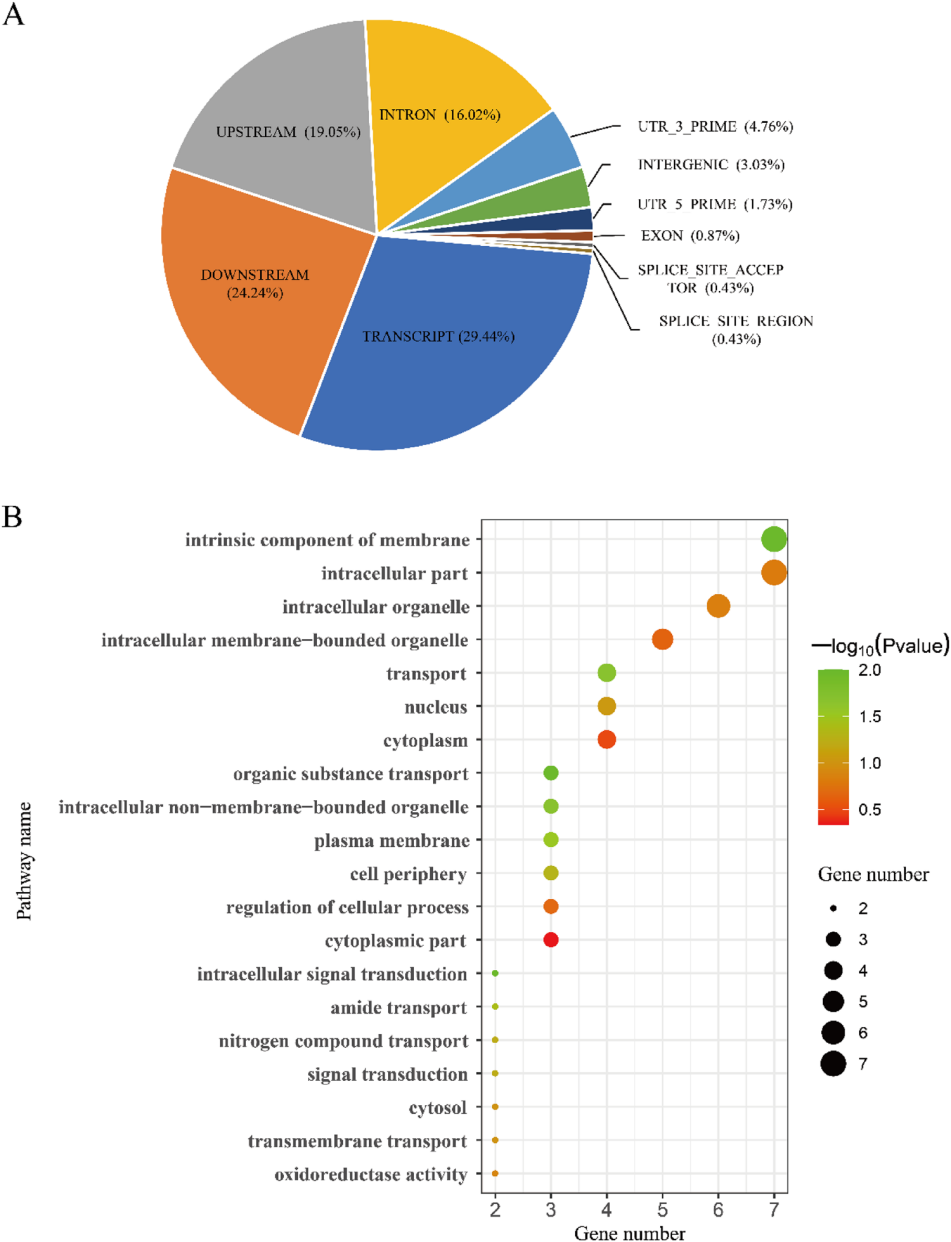
(**A**) Gene annotation for significant SNPs. (**B**) Top 20 reached Gene Ontology terms found by functional enrichment analysis. A total of 30 genes were mapped through 18 significant markers identified by GWAS analysis, with 48.49% positioned on transcript and upstream regions. Most of these genes are involved on the pathways of “biological process”, six of them were related to nitrogen compound metabolic processes.

**Figure 5:**
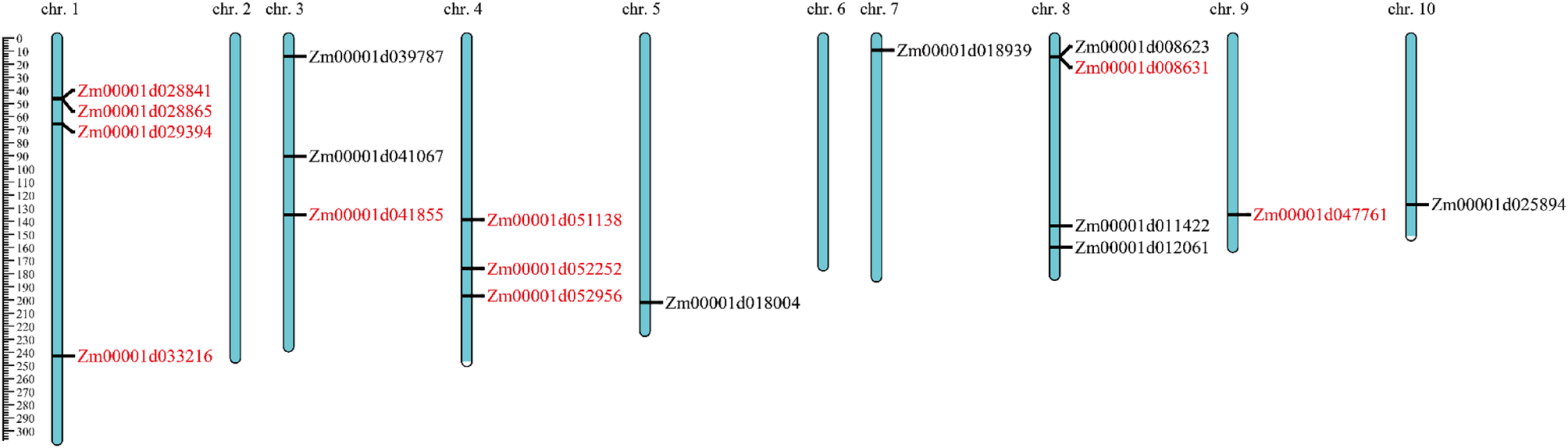
Distribution of candidate genes identified by GWAS across maize genome (RefGen B73 v.4). In chromosome 10 is possible to see the candidate gene (Zm00001d025894/AMT5) with a very well knowing function on nitrogen transportation transmembrane. *Note*: tandem duplications were indicated by red font.

Most of the significant SNPs were annotated as transcript (29.44%), downstream (24.24%), upstream (19.05%), or intron variants (16.02%), fewer than 12% of SNPs were annotated as utr_3_prime, intergenic, utr_5_prime, exon, splice_site_region, and splice_site_acceptor genomic regions (Fig. 4A). The top 20 ontology terms for genes near significant markers are shown in Fig. 4B, eleven of these genes are involved in cell composition and nine of them are involved in "biological process" pathways. In total, 30 genes were reached by the 18 significant SNPs, 6 of those candidate genes were related to nitrogen compound metabolic process (see Supplementary Table S3 online), one of them (Zm00001d051139) was reached by a marker (4582532|F|0–24:C > G-24:C > G) associated with SBNI and BNIPP. From the 10 SNPs significant for BNIPP and SBNI simultaneously, two were annotated as 5_prime_UTR_variant (Zm00001d039787 and Zm00001d029394), two as downstream_gene_variant (Zm00001d052252 and Zm00001d051138), one as upstream_gene_variant (Zm00001d041855), one as splice_region_variant & intron_variant (Zm00001d047761), one as missense_variant (Zm00001d011422), one as synonymous_variant (Zm00001d041067), one as intergenic_region (Zm00001d033216), and one as 3_prime_UTR_variant (Zm00001d018004). Ten genes were tandem duplications (Fig. 5), four of which were on chromosome 1, three in chromosomes 4, while chromosomes 3, 8 and 9 each had one duplicated gene.

## Discussion

Crops vary for BNI-capacity, and many plant species have some levels of BNI-activity in their root systems. BNI-capacity within a crop could be influenced by both genetics and its interaction with the environment^56^. Genotypes with large BNI-capacity typically release three to five times more BNI-activity from roots than low-BNI genotypes^15^. Several cereals, including pearl millet, sorghum, rice, barley, wheat, and maize, were initially reported to have little BNI-capacity (based on evaluation of only one genetic stock for each cereal)^16^. A wild relative of wheat (*Leymus racemosus* (Lam.) Tzvelev) released about 20-times more BNI-activity than root system of cultivated wheat^16^. Three elite sorghum cultivars (Hybridsorgo, IS41245, and GDLP 34-5-5-3) differed in their production of sorgoleone, a major hydrophobic BNI component; GDLP 34-5-5-3 and Hybridsorgo showed more sorgoleone release and hydrophobic BNI-capacity than IS41245^57^. In maize, four BNI-compounds were identified from hydrophobic BNI-activity, of which ‘zeanone’ was the dominant inhibitor^23^. Here, we have documented substantial genetic variation for hydrophobic BNI-capacity (SBNI, BNIPP, zeanone intensity) in a set of 250 CMLs. Consistent with previous research, we found a range of expression for the hydrophobic BNI-components, with only 10% of the CMLs having high BNI-capacity, with maxima reaching 56.35 ATU plant^−1^ for BNIPP, 1,694 ATU g^−1^ root dwt for SBNI, and 788,584 zeanone intensity for the released zeanone.

**Figure 6:**
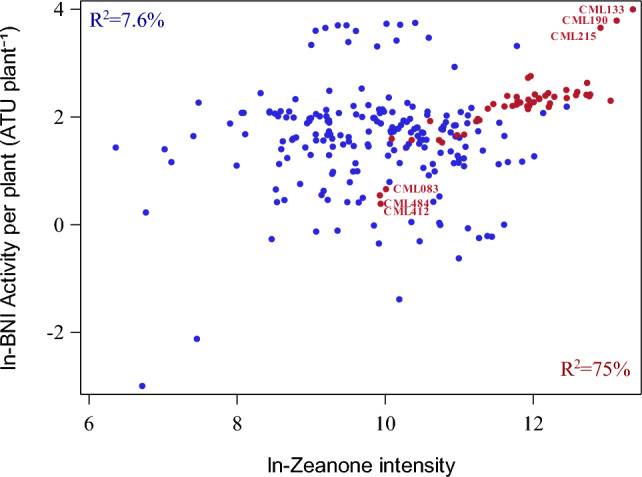
Linear simple regression of the CML mean natural logarithms of BNI per plant (BNIPP) with Zeanone intensity. R^2^: Coefficient of determination (%). The relationship between ln Zeanone and ln BNIPP when only 50 CMLs (red, batch 2) are included in the analysis is higher than when are used all 250 CMLs (blue + red, batch 1 and batch 2). Correlation between Zeanone intensity and BNIPP was larger for quality protein maize lines (r = 0.62, filled circle) than non-QPM lines (r = 0.23, empty triangle), CML without QPM information are represented with empty squares.

Maize productivity remains below its potential, due partly to inappropriate N management (e.g. N rate and time of applications)^58,59^. Nitrogen losses from maize production systems are largely due to nitrifier-activity and rapid generation of soil-nitrates, responsible for low nitrogen recovery and NUE in maize farming^60,61^. Use of nitrification inhibitors (such as dicyandiamide) to reduce nitrogen losses in maize farming and to improve soil-NH_4_^+^ levels has been recommended^62,63^; however, lack of consistency and high costs of synthetic nitrification inhibitors has limited their adoption^4^. Because plant BNI-activity delivers nitrification inhibitors from root systems directly to soil nitrifier-microsites where nitrifier-activity is concentrated, it is practical and economical^6^ to genetically exploit BNI-traits to develop maize cultivars that can regulate soil nitrifier-activity and limit soil-nitrate formation. Recently it was reported that zeanone, an important hydrophobic BNI-compound, contributed 28% of the hydrophobic BNI-capacity in maize cultivar cv. ‘Peter Corn’^23^. For our set of 250 CMLs, however, only 7.6% of the variation for BNIPP was explained by zeanone intensity (Fig. 6); however, in a sub-set of 50 CMLs (from these 250 CMLs), zeanone levels explained nearly 75% of variation in BNIPP, suggesting that other variants of zeanone (i.e. isomers or precursors of zeanone derivatives, that may have BNI-function) may exist in these inbred lines, but were not detected by our analytical methodology, i.e. zeanone derivatives may have BNI-activity but not detected and quantified by the HPLC-mass spectrometric analysis. Variation in root development and differences in dry weight of roots among CMLs could further affect the values obtained during zeanone measurement on the root surface. It is important to highlight that the current results are based on laboratory evaluations, which indicate potential for developing BNI-enabled maize in future.

The CMLs originated from diverse germplasm sources, and their genetic diversity, which was confirmed previously by molecular markers^64^, could include variation for BNI-activity. Interestingly, although we have no explanation, the correlation between zeanone intensity and BNIPP was larger (r = 0.62, n = 34, *P* < 0.0001) for quality protein maize (QPM)^65^ germplasm than non-QPM germplasm (r = 0.23, n = 208, *P* < 0.0001). Similar values were found for the correlation between zeanone intensity and SBNI, which was larger for QPM (r = 0.61, n = 34, *P* = 0.0001) than for non-QPM lines (r = 0.25, n = 208, *P* = 0.0002). Identifying additional compounds contributing to hydrophobic BNI-activity in the rhizosphere may be crucial to understanding these relationships and to design and implement breeding programs for increased BNI-activity of root systems. The hydrophobic BNI-capacity in maize appears to be stronger (5 to 50 ATU plant^−1^) than reported for wheat (5 to 10 ATU plant^−1^), and similar to that in sorghum, which has one of the strongest reported hydrophobic BNI-capacities^3^.

Genome-wide association study (GWAS) is cost- and time-efficient because there is no need to generate specific mapping populations to find associations between genotypic variations and phenotype measurements^49,66^. The GWAS analysis using 182,252 SNPs, extracted from another CML panel, identified 5 and 6 significant SNPs for grain yield under optimum and low-N conditions, respectively^53^. Also related to N use in maize, a GWAS for an IMAS (Improved Maize for African Soils) panel identified 47 genomic regions strongly (2.5 × 10^−6^ ≤ *P* ≤ 9.9 × 10^−4^) associated with grain yield under low N conditions, explaining from 3.4 to 10.6% of the total phenotypic variation^67^. In this study, we found 18 markers significantly associated with hydrophobic BNI-capacity and one of them linked exclusively to zeanone activity. Understanding the processes causing BNI-effects and identification of genes that increase their expression in maize could be useful for improving performance in regions with low soil fertility or limited access to nitrogen fertilizers. Molecular markers linked to genes or functional loci associated with BNI-capacity could be used in breeding programs, as previously shown for NUE^53^. Currently, there are no precedents for using molecular markers for BNI in maize or any other crop. Further, we identified 30 candidate genes potentially associated with BNI-activity. The marker 100063204|F|0–36:T > A-36:T > A, associated with SBNI-activity and located on chromosome 10 (133240300 bp), is in the region of Zm00001d025894 (also known as AMT5), which has been annotated as a protein coding gene involved in the regulation of ammonium transportation through cell membranes. This gene family (ZmAMT1;1a and ZmAMT1;3) was previously reported to be involved in substrate-inducible regulation of ammonium uptake in maize roots^68^. An appropriate proportion of ammonium may boost maize root proliferation and improve N nutrient uptake, which could have an impact on fertilizer use efficiency^69^. In addition, ammonium uptake and assimilation were reported to stimulate synthesis and release of BNI-substances from roots of wheat, sorghum and brachiaria^7,16,18,70^. Some of the genes we identified as possibly associated with BNI-activity have other important functions. For example, the putative gene Zm00001d018004 (also known as Rop1), was enriched by the SNP (2466232|F|0–67:C > T-67:C > T) with the greatest effect on SBNI and BNIPP simultaneously in this study. Rop1 plays an important role in the adaptation of plants to different environmental situations^71^, it has also influences on the infection capacity of sugarcane mosaic virus (SCMV) in maize plants; enhancing the infection in maize plants when it is silenced and decreasing it with its overexpression^72^. Marker 4582532|F|0–24:C > G-24:C > G has been also significant for both, SBNI and BNIPP, and has enriched the putative gene Zm00001d051139 (umc1702), which is involved in the directed movement of nitrogen-containing compounds between cells (see Supplementary Table S3 online), and linked also with RPi1, a gene that confers resistance to stalk rot in maize, placing the resistance gene on chromosome 4^73^. As we gain more insights about interactions between expression of BNI-trait and soil water status^74^, the pleiotropic relationships among genes linked with BNI-function and other gene’s expression will become clearer. We did not identify any genomic region clearly associated with the evaluated BNI-raits for our CML panel. All significant markers found in this study had LD decay distances between them greater than 1Mbp, this could have been due to the germplasm evaluated in this study. The limited, but still existing, genetic diversity of these inbred lines has an impact on the number of markers obtained, which also has a relationship with LD decay, once this a population-based function^75^. Nevertheless, the wide and uniform distribution of SNPs throughout the entire genome has revealed significant markers that are distant from each other. Our results achieved an important first step towards using genomic tools to identify markers for use in breeding for enhanced BNI-function in maize. However, much research is needed to introduce BNI-capacity into high-yielding commercial hybrids before the true potential of BNI-enabled maize can be realized when deployed in production agriculture. Only future research developments can answer the real implications from this research.

The identification of 18 SNPs associated with hydrophobic BNI-activity, suggests the potential to implement MAS or GS in breeding for nitrification inhibition in maize. In addition, the detection of 6 candidate genes related to NUE shows that our GWAS analysis identified genomic regions associated with N-related traits; with special attention on the SNP that has been locating in the region of AMT5, a protein coding gene involved in the regulation of ammonium transportation. Our results were obtained from a panel of elite inbred lines (CMLs), which was not a mapping population developed for BNI-activity. We are currently developing four doubled haploid (DH) maize populations from crosses between CMLs identified here with contrasting hydrophobic BNI-capacity and zeanone activity. This will be a valuable next step to generate genetic markers for possible use in breeding for enhanced BNI-activity in maize. Another complex area of urgently needed research is to develop and validate high throughput methods to measure hydrophobic and hydrophilic BNI-activity for plants in the field rather than grown in glasshouses.

## Conclusions

We are in a “discovery phase” for BNI in maize. This study identified significant variation among CMLs for hydrophobic BNI-capacity, with some CMLs having tenfold greater capacity than others, and some CMLs having high levels of zeanone intensity, a major hydrophobic BNI-component released from maize roots. Other key findings from this study include: (1) identification of 18 SNPs associated with hydrophobic BNI-activity, suggesting potential to implement GS or MAS in breeding for nitrification inhibition in maize; (2) the detection of 6 candidate genes related to NUE shows that our GWAS analysis identified potential genomic regions associated with N-related traits; (3) significant markers were observed in genomic regions with low signal and high LD decay distance (> 1Mbp), suggesting the absence of selection for these traits in the CMLs, 4) Exposure of a gene with known functions in the regulation of ammonium transport across the cell membrane. The ongoing creation of doubled haploid lines from crosses of high by low BNI-activity elite inbred lines will allow scientists to develop a specific set of markers that could help in a potential breeding process of BNI in maize.

## Methods

### Building a core germplasm set

All experimental research was conducted in alignment with CIMMYT policies and procedures (Guidelines and policies – CIMMYT). In our study the sample materials used are inbreed lines developed as international public goods by CIMMYT. These materials were sourced from the CIMMYT genebank, where materials are conserved in trust and shared under the Standard Material Transfer Agreement (SMTA) of the International Treaty on Plant Genetic Resources for Food and Agriculture.

We used genotypic data (www.seedsofdiscovery.org) developed for 475 CIMMYT maize inbred lines (CMLs), available in https://data.cimmyt.org/, to select 250 CMLs with seeds available from CIMMYT’s germplasm bank (see Supplementary Table S4 online). We used Franco et al.’s^76,77^ 2-stage-D method in which: (1) the allele frequencies from 169,061 SNP-DarTseq markers with fewer than 50% of missing values, and the modified Rogers genetic distance (MRD)^78^ between every pair of lines were calculated, and (2) the CMLs were clustered based on the MRD, using Ward’s hierarchical method^79^. Eleven groups were identified, and their average MRD values were calculated. The number of lines to be selected from each group was defined, proportionally to their diversity, and 1,000 possible subsets were identified by a stratified random sampling procedure. The average diversity value for each candidate subset was calculated and the subset of 250 CMLs with the largest diversity was selected. Minimum, mean, and maximum diversity values were calculated for the whole collection and the selected core subset (see Supplementary Table S5 online). More information about CMLs can be found at https://hdl.handle.net/11529/10246.

### Phenotyping of hydrophobic-BNI

#### Raising plants

Hydrophobic BNI-capacity was assessed using a modified method developed originally for sorghum^18,80^; which has been described in detail previously^23^. Ninety seeds were soaked in an Eppendorf tube (50 mL vol) with 40 mL aerated distilled water in darkness at 25 °C for 24 h (h). The soaked seeds were wrapped with a wet towel to allow sprouting for another 24 h. The germinated seeds were arranged in a folded cliff of a filter paper, supported by hard plastic plates with a rubber band, and kept in a growth box containing 200 mL of 200 mM CaSO4 aqueous solution at the bottom; the capillary force kept the seeds in the cliff fold of the filter paper moist during the seedling growth period. The seedling-grow-boxes were kept on racks in a growth chamber, with fluorescent lighting with a photoperiod of 13/11 h (day/night). Roots of twelve-day-old seedlings were used for assessing hydrophobic BNI-activity, using two replications of 20 seedlings for each genotype (CML).

### Collecting root-DCM wash for hydrophobic BNI-activity determinations

Twenty seedlings were randomly selected, their roots were separated and dipped in 50 mL of 1% acidified DCM (dichloromethane; v/v) for 60 s (sec) before removing and drying the roots using paper towels. The roots were then oven-dried at 70 °C for 48 h before measuring their dry weight. The DCM wash was filtered and evaporated in vacuum at 40 °C, and the residue was extracted with 20 mL methanol. The methanol extract was evaporated and condensed to 100 mL of methanol; this methanol extract was evaporated to dryness, and extracted with DMSO (dimethylsulfoxide), which was used for determining BNI-activity using bioluminescence assay described below.

### Nitrification inhibition bioassay

The methanol extract was evaporated and extracted with 20 µL dimethylsulfoxide (DMSO); 1 µL of this sample is used to determine BNI-activity using the luminescent recombinant *Nitrosomonas* described earlier^8^. In brief, the recombinant strain of *N. europaea* expresses luciferases of *luxA* and *luxB* genes from the marine bacterium *Vibrio harveyi* and produces a specific luminescence pattern with two distinct peaks during a 30-s measurement period. The key functional relationship between bioluminescence emission and NO_2_^−^ production is linear when using the synthetic nitrification inhibitor, allylthiourea (AT) as a standard. The inhibition caused by AT of 0.22 μM in the assay medium, ED_80_ in bioluminescence and NO_2_^−^ production, is defined as 1 allylthiourea unit (ATU). The inhibitory activities of root extracts, root exudates and compounds are expressed in ATU based on dose–response standard curve of AT. The biological nitrification inhibitory activities in this study were expressed as per plant (BNIPP [ATU plant^−1^]), where the obtained inhibitory activity was divided by number of seedlings used, and the specific activity (SBNI [ATU g^−1^ root dry wt.]), where the inhibitory activity was calculated per mass of root-DCM wash.

### Determination of zeanone using LCMS

The methanol extract was analyzed for zeanone according to Otaka et al.^23^. Semi-quantification was achieved as follows, an aliquot of the methanol extract (5 uL) was analyzed by Shimadzu LCMS-2020 equipped with DUIS-2020 on a TSKgel Super-ODS [10% acetonitrile (0 min), 50% acetonitrile (8 min) then 100% acetonitrile (10 min), 0.4 mL/min]. Zeanone was detected with a mass spectrometric detection in positive mode, single ion monitoring with m/z 219 representing zeanone with 0.5 s event time. Semi-quantification was achieved based on the peak area eluted at 528 s, representing zeanone. The peak area indicates relative content of zeanone in the sample, but not the absolute content; therefore, we refer to the peak area as zeanone intensity.

### Statistical description of phenotypic data

Phenotypic data from 250 CMLs can be found as Supplementary Table S6 online, it has been used to calculate mean, median, standard deviation, coefficient of variation, minimum and maximum for statistical description. A histogram for each response shows the potential skewed distribution of the data. CML means from phenotypic data were obtained by a linear mixed model for a completely randomized design with two replicates. Responses to variables were transformed for further analysis using natural logarithm because of the skewed distribution of raw data. A similar model that considers CML effect as random was fitted to estimate genetic variance and broad sense heritability.

### Association analysis for hydrophobic BNI-activity

CIMMYT’s Seed of Discovery project^81^ used DArTseq technology (www.diversityarrays.com) to characterize the 28,000 CIMMYT maize germplasm collections, including elite inbred lines CMLs^82^. This method is based on a complexity reduction process where a combination of two enzymes is implemented to generate a genome representation, followed by sequencing of the resulting fragments^83^. A proprietary analytical pipeline developed by DArT P/L was used to generate allele calls for SNP (co-dominant) and Silico-DArT (dominant) markers from the same assays^84,85^. The same pipeline was used to develop a new SNP calling exclusively for the 250 CMLs that were evaluated in this study. A matrix was developed, with DArTseq-SNP markers in the rows, and genotypes in the columns, where the genotypic data scoring represents the dominant homozygous as 0, recessive homozygous as 1 and the heterozygote as 2, according to Petroli and Kilian^84^. In this study we decided to replace the heterozygote genotypes (score 2) by a missing data score (-) because we are working with homozygous lines and to its small representation (1.6%) in our data set. A set of filtering parameters (e.g., One Ratio, Call Rate and Reproducibility) was then applied. Marker sequences were blasted against the *Zea mays L*. reference genome (B73 RefGen_v4), and only SNPs with a unique physical position were considered in further analyses. An additional marker selection criterion was applied, where monomorphic markers and those in which only one CML presented a different allele were removed from analyses. Based on these criteria, from a total of 56,411 SNPs obtained, we selected 27,085 high quality polymorphisms for the GWAS analysis (see Supplementary Table S7 online).

For the association analysis we included in the analytical model the dimensions of the principal component analysis. We tested the models using 0, 2, 3, 5, and 10 principal dimensions, then for each model we had two different models, one without the genetic relationship between CMLs, and another model considering the genetic relatedness between them. Comparison between models was performed considering the Bayesian Information Criterion (BIC) and q-q plots of p-values. Based on these parameters, the best models did not include principal dimensions for SBNI and BNIPP but included the kinship matrix, while for Zeonane the best model did not include neither. GWAS was performed using the following linear model using Tassel 5^86^.

$$g_{i} = {\text{m}} + { }\mathop \sum \limits_{j = 1}^{p} \alpha_{j} x_{ji} + \beta_{{\varvec{i}}} + e_{{\varvec{i}}}$$

where ${g}_{i}$ is the log_10_ adjusted phenotypic observation of the *i*th CML; m is the overall mean, $\sum_{j=1}^{p}{\alpha }_{j}{x}_{ji}$ is the effect of the *j*th first principal components (*P* = 0, 2, 3, 5, 10), ${\beta }_{i}$ is the fixed effect of a single marker between the two homozygous genotypes, and ${e}_{i}$ is the vector of random residual error, $e\sim N(0,{\sigma }^{2}{\varvec{I}})$, where *I* is the identity matrix. We considered an additional model in which $e\sim N(0,{\sigma }^{2}{\varvec{K}})$, where ***K*** is the kinship relationship obtained from marker data. Significance values were adjusted by FDR (−Log10) method^87^ to reduce the rate of false positives. R-square was calculated for significant markers to evaluate the importance of the marker effect.

### Gene annotation and enrichment

To measure the linkage disequilibrium (LD) between GWAS-significant markers we calculated the standardized regression coefficient (SRC) at a maximum distance of 1Mbp. LD decay curves were estimated by chromosome, and the SRC between SNPs were calculated. Within those windows, we traced putative genes that could be related to processes involving the participation of nitrogen as an essential element.

We used SnpEff software to conduct functional annotations and predictions for the target SNPs^88^. The maize B73v.4 gene annotation was downloaded as a gff3 file from the Maize Genetics and Genomics Database (MaizeGDB) (https://www.maizegdb.org/assembly). Functional enrichment analysis of the annotated genes was performed using the ClueGO plugin for cytoscape^89^, and its results were plotted with the R package ggplot2^90^. Mapchart software was used to plot gene distributions across the maize genome^91^.

### Supplementary Information

Supplementary Figure 1.

Supplementary Figure 2.

Supplementary Figure 3.

Supplementary Figure 4.

Supplementary Figure 5.

Supplementary Figure 6.

Supplementary Table 1.

Supplementary Table 2.

Supplementary Table 3.

Supplementary Table 4.

Supplementary Table 5.

Supplementary Table 6.

Supplementary Table 7.

## Data Availability

The datasets generated during and/or analyzed during the current study are available in the CIMMYT Dataverse repository, https://data.cimmyt.org/privateurl.xhtml?token=861d24b1-d3fc-49bb-95f2-a086986fb2de.
