# Supplementary material for: Genetic variation among elite inbred lines suggests potential to breed for BNI-capacity in maize
Source: Sci Rep. 2023 Aug 17;13:13422. doi: 10.1038/s41598-023-39720-3 (PMC10435450; doi:10.1038/s41598-023-39720-3)
Supplement: Supplementary file 1 — Supplementary Figure 1. [file 41598_2023_39720_MOESM1_ESM.docx]

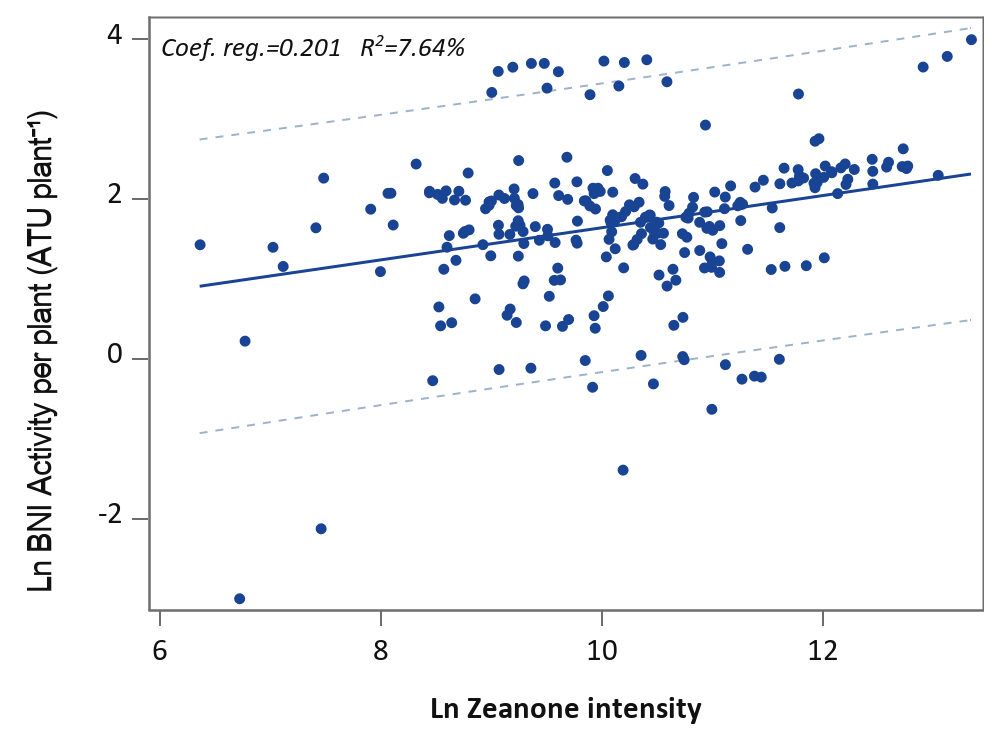


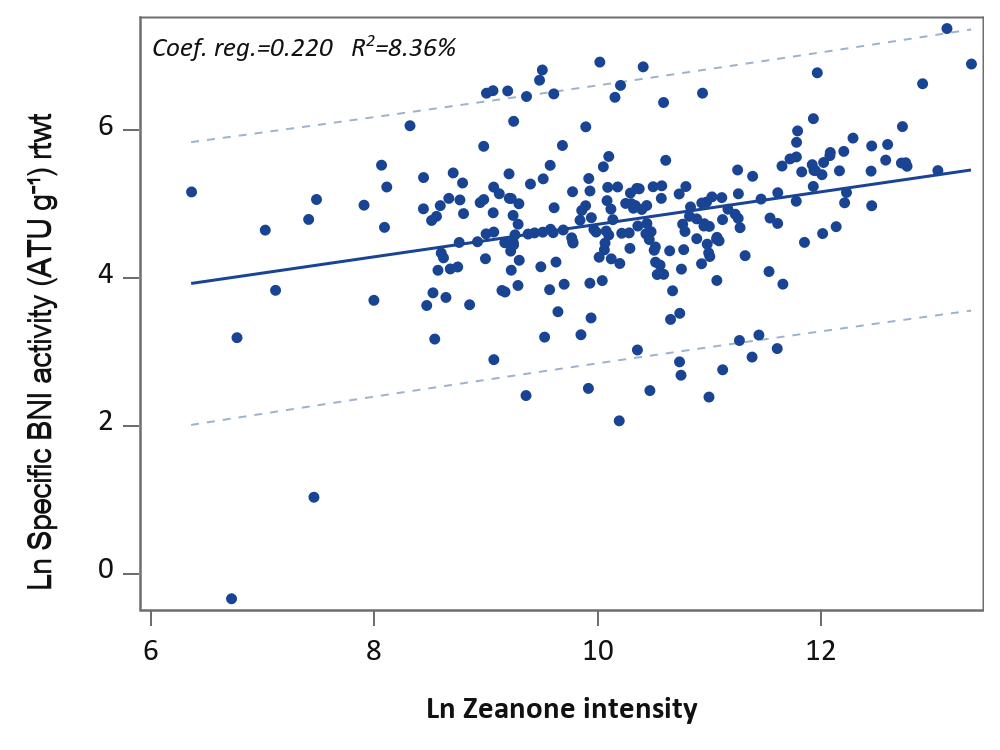


**Fig. S1** Linear simple regression of the CML mean natural logarithms of Zeanone with BNI per plant (BNIPP) and Specific BNI (SBNI). Dotted line is the 95% confidence interval for one individual (one CML). Coef. Re.: linear coefficient of regression, R^2^: square of the correlation coefficient
