# Supplementary material for: Genetic variation among elite inbred lines suggests potential to breed for BNI-capacity in maize
Source: Sci Rep. 2023 Aug 17;13:13422. doi: 10.1038/s41598-023-39720-3 (PMC10435450; doi:10.1038/s41598-023-39720-3)
Supplement: Supplementary file 2 — Supplementary Figure 2. [file 41598_2023_39720_MOESM2_ESM.docx]

**Fig. S2** Functional relationship between Total hydrophobic-BNI activity and zeanone levels in root-DCM wash of 50 maize CMLs (second batch) are high (R^2^ 0.63). Although the strength of the correlation may seem to be increased due to three extreme values, when they are removed from the analysis the correlation remains high (R^2^ 0.75)
