# Supplementary material for: Genetic variation among elite inbred lines suggests potential to breed for BNI-capacity in maize
Source: Sci Rep. 2023 Aug 17;13:13422. doi: 10.1038/s41598-023-39720-3 (PMC10435450; doi:10.1038/s41598-023-39720-3)
Supplement: Supplementary file 3 — Supplementary Figure 3. [file 41598_2023_39720_MOESM3_ESM.docx]

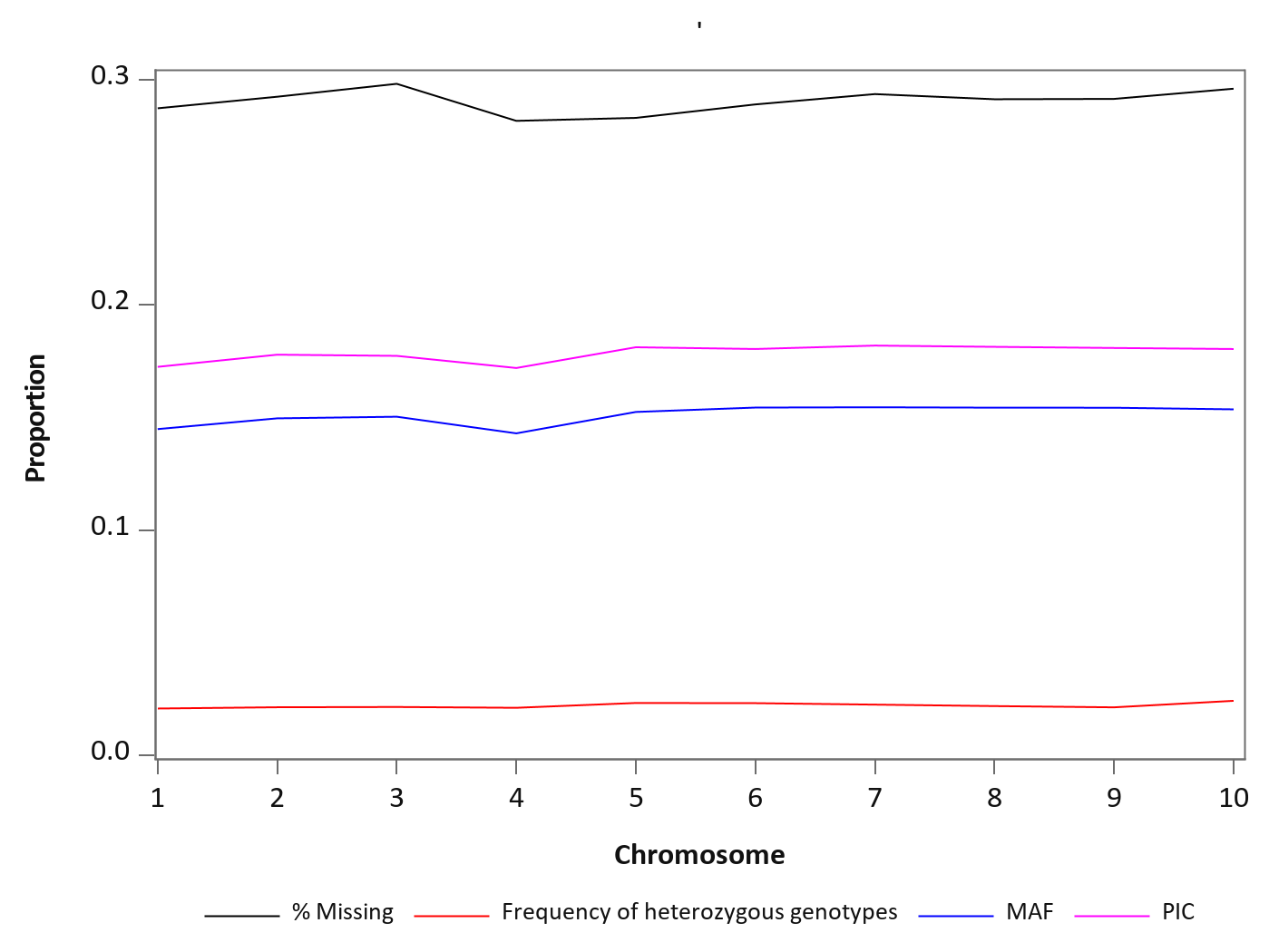


**Fig. S3** Characterization of molecular markers. Proportion of missing data (black), frequency of heterozygous genotypes (red), minor allele frequency (MAF) (blue), and polymorphic information content (PIC) (pink) per chromosome. Results confirm the low level of heterozygous loci, minor allele frequency and polymorphic information content in the set of lines used in this analysis
