# Supplementary material for: Genetic variation among elite inbred lines suggests potential to breed for BNI-capacity in maize
Source: Sci Rep. 2023 Aug 17;13:13422. doi: 10.1038/s41598-023-39720-3 (PMC10435450; doi:10.1038/s41598-023-39720-3)
Supplement: Supplementary file 4 — Supplementary Figure 4. [file 41598_2023_39720_MOESM4_ESM.docx]

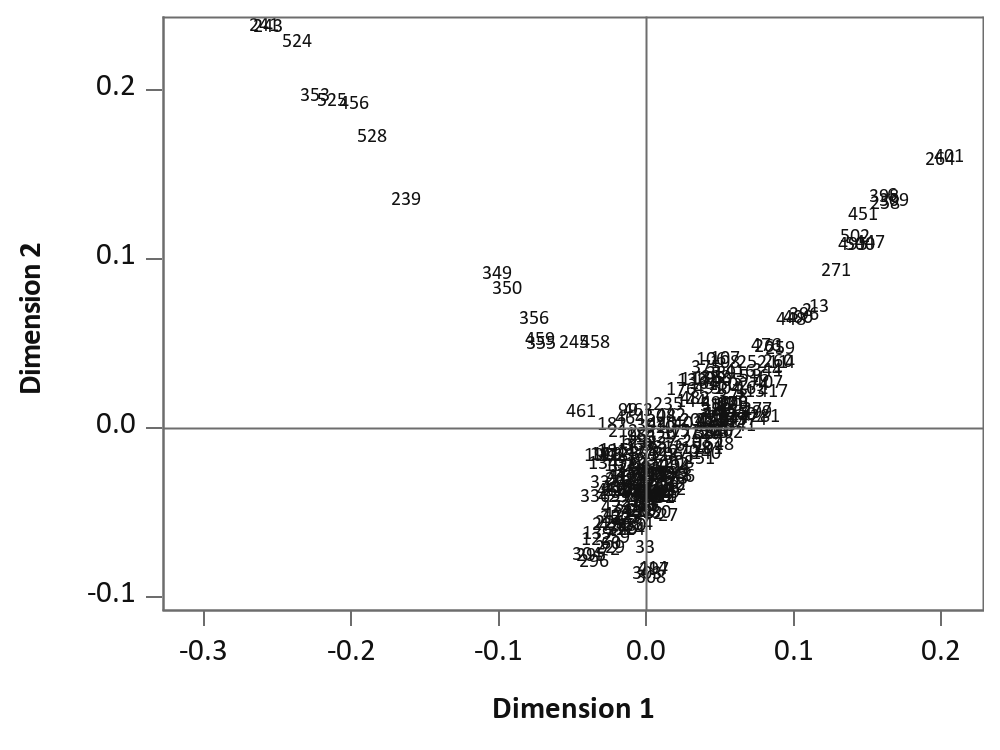


**Fig. S4** Two first dimensions of multidimensional scaling using representation of the modified Roger distance between 250 CMLs. Numbers represent the original number of each CML. There is not a clear clustering effect on the analyzed set of samples
