# Supplementary figures and images for: Genetic variation among elite inbred lines suggests potential to breed for BNI-capacity in maize
Source: Sci Rep. 2023 Aug 17;13:13422. doi: 10.1038/s41598-023-39720-3 (PMC10435450; doi:10.1038/s41598-023-39720-3)

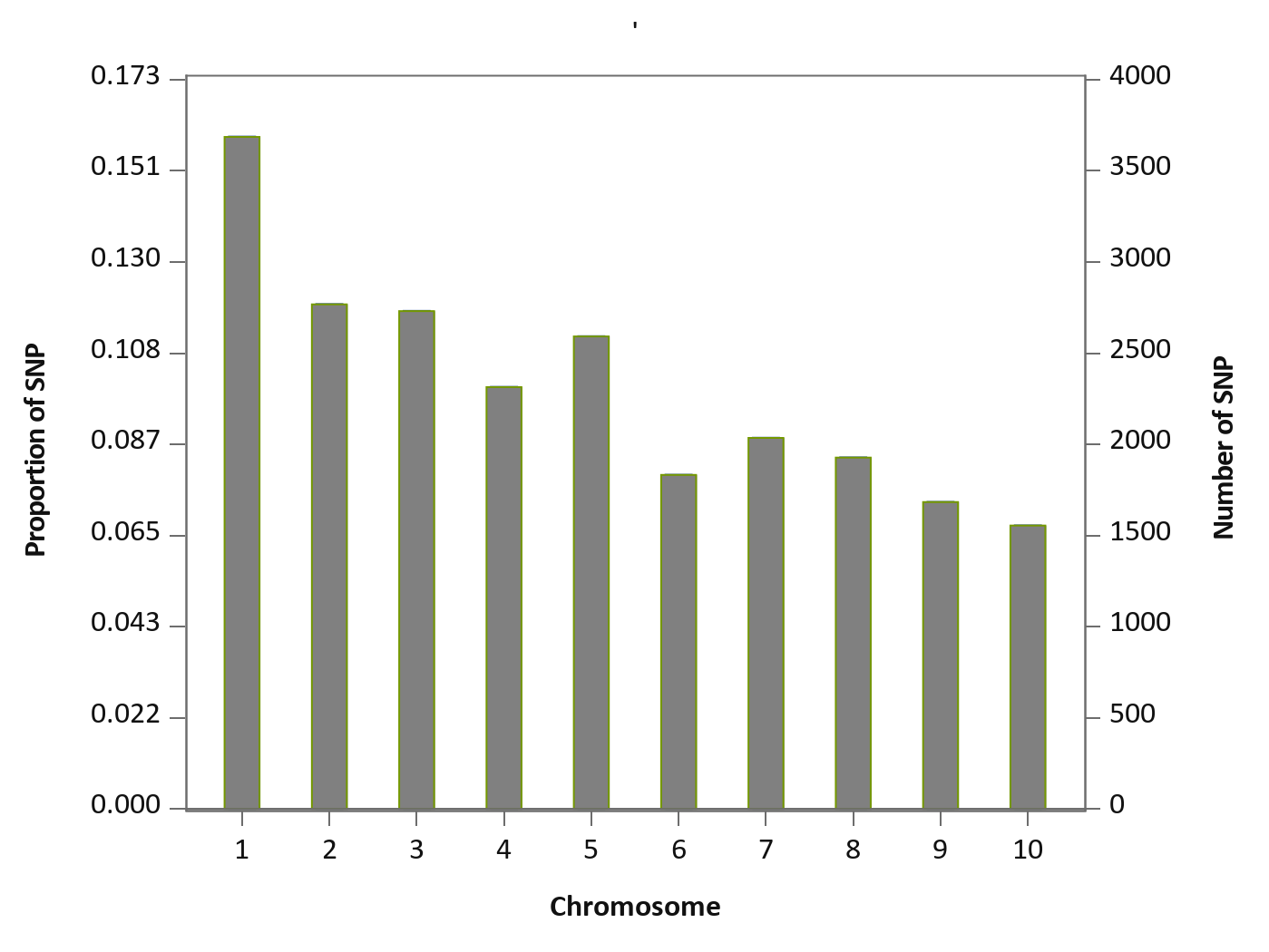


**Fig. S5** SNPs density on each chromosome of the reference genome (B73 RefGen v4)

Supplement: Supplementary file 5 — Supplementary Figure 5. [file 41598_2023_39720_MOESM5_ESM.docx]
