# Supplementary material for: Genetic variation among elite inbred lines suggests potential to breed for BNI-capacity in maize
Source: Sci Rep. 2023 Aug 17;13:13422. doi: 10.1038/s41598-023-39720-3 (PMC10435450; doi:10.1038/s41598-023-39720-3)
Supplement: Supplementary file 6 — Supplementary Figure 6. [file 41598_2023_39720_MOESM6_ESM.docx]

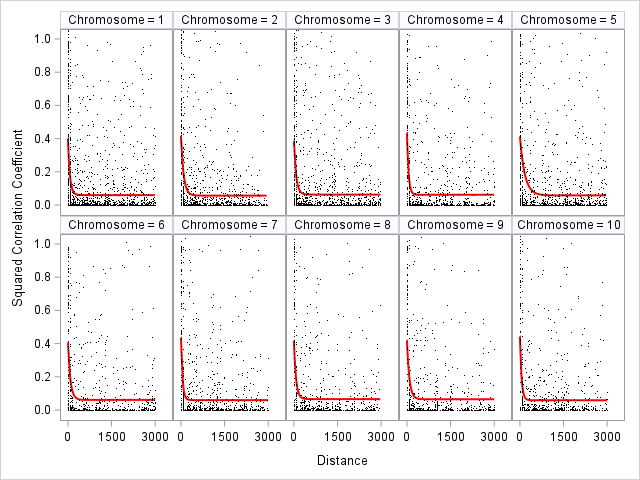


**Fig. S6** LD decay analysis by chromosome. The red line represents the fitted curve SRC=a+b*log(distance)+e. Distances are represented in Mbp.
