# Supplementary material for: Genetic variation among elite inbred lines suggests potential to breed for BNI-capacity in maize
Source: Sci Rep. 2023 Aug 17;13:13422. doi: 10.1038/s41598-023-39720-3 (PMC10435450; doi:10.1038/s41598-023-39720-3)
Supplement: Supplementary file 7 — Supplementary Table 1. [file 41598_2023_39720_MOESM7_ESM.docx]

| **Trait** | **Error DF** | **Genetic Variance** | **Residual Variance** | **-2LnLikelihood** | **BIC** | **Number of Parameters** | **Number of PCA Dimensions** | **Kinship matrix** |
| --- | --- | --- | --- | --- | --- | --- | --- | --- |
| LBNIPP | 247 | 0.24015 | 0.57235 | 668.40 | **684.96** | 3 | 0 | IBS |
| LBNIPP | 247 | 0.00075 | 0.90188 | 672.92 | 689.48 | 3 | 0 | Identity |
| LBNIPP | 245 | 0.27185 | 0.54040 | 663.05 | 690.66 | 5 | 2 | IBS |
| LBNIPP | 245 | 0.00075 | 0.90853 | 669.24 | 696.85 | 5 | 2 | Identity |
| LBNIPP | 244 | 0.28222 | 0.53063 | 661.37 | 694.50 | 6 | 3 | IBS |
| LBNIPP | 242 | 0.27289 | 0.53906 | 653.83 | 698.00 | 8 | 5 | IBS |
| LBNIPP | 242 | 0.00075 | 0.89861 | 659.37 | 703.54 | 8 | 5 | Identity |
| LBNIPP | 237 | 0.25599 | 0.55243 | 634.85 | 706.63 | 13 | 10 | IBS |
| LSBNI | 247 | 0.20376 | 0.70633 | 692.08 | **708.65** | 3 | 0 | IBS |
| LSBNI | 247 | 0.00082 | 0.98383 | 694.31 | 710.88 | 3 | 0 | Identity |
| LSBNI | 245 | 0.24887 | 0.65755 | 686.79 | 714.39 | 5 | 2 | IBS |
| LSBNI | 245 | 0.00082 | 0.99121 | 690.49 | 718.10 | 5 | 2 | Identity |
| LSBNI | 244 | 0.26312 | 0.64326 | 685.13 | 718.26 | 6 | 3 | IBS |
| LSBNI | 242 | 0.25598 | 0.65012 | 677.85 | 722.02 | 8 | 5 | IBS |
| LSBNI | 242 | 0.00082 | 0.98431 | 681.32 | 725.49 | 8 | 5 | Identity |
| LSBNI | 237 | 0.27978 | 0.62687 | 661.61 | 733.39 | 13 | 10 | IBS |
| LZeo | 250 | 0.38389 | 1.19055 | 839.22 | **839.22** | 0 | 0 | IBS |
| LZeo | 250 | 0.00144 | 1.74022 | 845.79 | 845.79 | 0 | 0 | Identity |
| LZeo | 248 | 0.39798 | 1.17707 | 831.49 | 842.53 | 2 | 2 | IBS |
| LZeo | 248 | 0.00144 | 1.73409 | 838.13 | 849.17 | 2 | 2 | Identity |
| LZeo | 247 | 0.38083 | 1.19097 | 825.47 | 842.04 | 3 | 3 | IBS |
| LZeo | 245 | 0.35721 | 1.20952 | 816.16 | 843.77 | 5 | 5 | IBS |
| LZeo | 245 | 0.00140 | 1.69053 | 820.76 | 848.36 | 5 | 5 | Identity |
| LZeo | 240 | 0.19732 | 1.34871 | 790.50 | 845.72 | 10 | 10 | IBS |

LBNIPP: Ln Biological Nitrification Inhibition Per Plant activity

LSBNI: Ln Specific Nitrification Inhibition Per Plant activity

LZeo: Ln Zeanone-intensity

IBS: Identical-by-State

**Supplementary Table S1.** Comparison between different models considering the relationship and not relationship between all the 250 CMLs observed, analysis was done using the Bayesian Information Criterion (BIC) method.
