# Supplementary material for: Genetic variation among elite inbred lines suggests potential to breed for BNI-capacity in maize
Source: Sci Rep. 2023 Aug 17;13:13422. doi: 10.1038/s41598-023-39720-3 (PMC10435450; doi:10.1038/s41598-023-39720-3)
Supplement: Supplementary file 10 — Supplementary Table 4. [file 41598_2023_39720_MOESM10_ESM.docx]

**Supplementary Table S4.** CIMMYT Maize Inbred Lines (CMLs), columns are showing the CMLs identification #; Population used to develop the each CML, environmental adaptation, and Maturity of each CML. More information about CMLs in <https://hdl.handle.net/11529/10246>. Interm (intermediate), NA (Not available)

| **CIMMYT Maize Lines** | | | | | | | |
| --- | --- | --- | --- | --- | --- | --- | --- |
| **CML** | **Source germplasm** | **Adaptation** | **Maturity** | **CML** | **Source germplasm** | **Adaptation** | **Maturity** |
| CML001 | P21 | Lowland | Late | CML274 | P43 | Lowland | NA |
| CML002 | P21 | Lowland | Late | CML275 | P43 | Lowland | NA |
| CML004 | P21 | Lowland | Interm | CML277 | P43 | Lowland | NA |
| CML006 | P21 | Lowland | Late | CML279 | P43 | Lowland | NA |
| CML011 | P21 | Lowland | Late | CML281 | P43 | Lowland | NA |
| CML013 | P21 | Lowland | Late | CML282 | G22 | Lowland | NA |
| CML015 | P22 | Lowland | Late | CML284 | G22 | Lowland | NA |
| CML016 | P22 | Lowland | Late | CML286 | P24 | Lowland | NA |
| CML017 | P22 | Lowland | Late | CML287 | P24 | Lowland | NA |
| CML019 | P24 | Lowland | Late | CML289 | P24 | Lowland | NA |
| CML021 | P24 | Lowland | Interm | CML292 | P28 | Lowland | NA |
| CML022 | P25 | Lowland | Interm | CML295 | SintAmTSR | Lowland | NA |
| CML023 | P25 | Lowland | Interm | CML296 | SintAmTSR | Lowland | NA |
| CML025 | P26 | Lowland | Interm | CML297 | SintAmTSR | Lowland | NA |
| CML027 | P27 | Lowland | Interm | CML303 | SintAmTSR | Lowland | NA |
| CML029 | P27 | Lowland | Late | CML304 | SintAmTSR | Lowland | NA |
| CML030 | P27 | Lowland | Late | CML308 | SintAmTSR | Lowland | NA |
| CML031 | P27 | Lowland | Late | CML309 | P43 | Lowland | NA |
| CML032 | P28 | Lowland | Interm | CML312 | P500 | Subtropical | Interm |
| CML033 | P28 | Lowland | Interm | CML313 | P501 | Subtropical | Late |
| CML035 | P29 | Lowland | Late | CML320 | P501 | Subtropical | Late |
| CML041 | P36 | Lowland | Interm | CML322 | REC | Subtropical | Interm |
| CML048 | P73 | Lowland | Late | CML323 | P33 | Subtropical | Interm |
| CML049 | P73 | Lowland | Interm | CML328 | REC | Subtropical | Early |
| CML050 | P78 | Lowland | Interm | CML329 | REC | Subtropical | Interm |
| CML051 | P79 | Lowland | Late | CML331 | REC | Subtropical | Early |
| CML055 | G24 | Lowland | Late | CML332 | REC | Subtropical | Early |
| CML056 | G24 | Lowland | Late | CML333 | P590 | Subtropical | Early |
| CML059 | Antigua | Lowland | Late | CML336 | REC | Subtropical | Early |
| CML060 | Antigua | Lowland | Late | CML337 | REC | Subtropical | Early |
| CML066 | P25 | Lowland | Late | CML340 | P43 | Lowland | NA |
| CML072 | Antigua | Lowland | Late | CML341 | P43 | Lowland | NA |
| CML076 | G32 | Subtropical | Interm | CML344 | P21 | Lowland | NA |
| CML082 | G32 | Subtropical | Early | CML345 | P390 | Lowland | NA |
| CML083 | G32 | Subtropical | Interm | CML348 | G26 | Lowland | NA |
| CML084 | G32 | Subtropical | Interm | CML349 | P36 | Highland | Early |
| CML089 | P34 | Subtropical | Interm | CML350 | NA | Highland | Early |
| CML096 | P42 | Subtropical | Interm | CML353 | NA | Highland | Early |
| CML099 | P42 | Subtropical | Interm | CML355 | P800 | Highland | Early |
| CML104 | P44 | Subtropical | Interm | CML356 | P800 | Highland | Early |
| CML105 | P44 | Subtropical | Interm | CML358 | SA3 | South America | Interm |
| CML106 | P44 | Subtropical | Late | CML359 | SA3 | South America | Interm |
| CML107 | P44 | Subtropical | Late | CML362 | SA5 | South America | Late |
| CML108 | P44 | Subtropical | Interm | CML365 | SA8 | South America | Interm |
| CML109 | P47 | Subtropical | Interm | CML369 | P402 | Subtropical | Early |
| CML113 | P33 | Subtropical | Interm | CML370 | MBRET | Subtropical | Interm |
| CML115 | P45 | Subtropical | Interm | CML371 | MBRET | Subtropical | Interm |
| CML117 | REC | Subtropical | Late | CML374 | P43SR | Subtropical | Interm |
| CML118 | SIYF | Subtropical | Late | CML375 | P44C9 | Subtropical | Interm |
| CML120 | NA | Subtropical | Late | CML377 | REC | Subtropical | Interm |
| CML122 | NA | Subtropical | Interm | CML378 | SLWHGB | Subtropical | Interm |
| CML123 | P47 | Subtropical | Interm | CML379 | P501 | Subtropical | Late |
| CML124 | P47 | Subtropical | Early | CML384 | P502 | Subtropical | Late |
| CML128 | G31 | Subtropical | Interm | CML386 | ZM601 | Africa MA/ST | Late |
| CML130 | P33 | Subtropical | Interm | CML388 | ZM609 | Africa MA/ST | Late |
| CML133 | P44 | Subtropical | Late | CML389 | ZM609 | Africa MA/ST | Late |
| CML134 | P45 | Subtropical | Interm | CML393 | REC | Africa MA/ST | Late |
| CML136 | P47 | Subtropical | Interm | CML396 | P21 | Lowland | Late |
| CML139 | NA | Subtropical | Interm | CML398 | P21 | Lowland | Late |
| CML140 | P62 | Lowland | Late | CML400 | P21 | Lowland | Late |
| CML144 | P62 | Lowland | Late | CML401 | P21 | Lowland | Late |
| CML145 | P63 | Lowland | Late | CML402 | P22 | Lowland | Late |
| CML147 | P63 | Lowland | Late | CML403 | P22 | Lowland | Late |
| CML148 | G23Q | Lowland | Late | CML405 | P43 | Lowland | Late |
| CML150 | G24Q | Lowland | Late | CML406 | P43 | Lowland | Late |
| CML151 | P62 | Lowland | Late | CML407 | G24 | Lowland | Late |
| CML152 | P62 | Lowland | Late | CML410 | P27 | Lowland | Late |
| CML157 | P62 | Lowland | Late | CML411 | P28 | Lowland | Late |
| CML158 | P62 | Lowland | Late | CML412 | P36 | Lowland | Late |
| CML159 | P63 | Lowland | Late | CML414 | SintAmTSR | Lowland | Late |
| CML163 | G26Q | Lowland | Late | CML416 | P49 | Lowland | Early |
| CML164 | P65 | Lowland | Late | CML417 | P49 | Lowland | Early |
| CML165 | P66 | Lowland | Late | CML419 | G15 | Lowland | Early |
| CML167 | G25Q | Lowland | Late | CML421 | P31 | Lowland | Early |
| CML172 | G25Q | Lowland | Late | CML422 | G17 | Lowland | Early |
| CML173 | P68 | Subtropical | NA | CML423 | G18 | Lowland | Early |
| CML175 | P68 | Subtropical | NA | CML425 | P31 | Asia Lowland | Early |
| CML176 | REC | Subtropical | NA | CML429 | EYDMR | Asia Lowland | Early |
| CML177 | G32Q | Subtropical | NA | CML432 | KTX3752 | Asia Lowland | Early |
| CML178 | G32Q | Subtropical | NA | CML436 | SA3 | South America | Late |
| CML181 | NA | Subtropical | NA | CML438 | SA4 | South America | Late |
| CML182 | NA | Subtropical | NA | CML440 | G16 | Africa MA/ST | Early |
| CML184 | G32Q | Subtropical | NA | CML442 | REC | Africa MA/ST | Interm |
| CML186 | P67 | Subtropical | NA | CML444 | P43 | Africa MA/ST | Late |
| CML190 | G34Q | Subtropical | NA | CML447 | P43 | Lowland | Late |
| CML191 | G34Q | Subtropical | NA | CML448 | P21 | Lowland | Late |
| CML192 | G34Q | Subtropical | NA | CML449 | P32 | Lowland | Interm |
| CML199 | na | Africa MA/ST | Late | CML451 | REC | Lowland | Late |
| CML201 | na | Africa MA/ST | Late | CML452 | P28 | Lowland | Interm |
| CML203 | na | Africa MA/ST | Late | CML453 | P24 | Lowland | Late |
| CML204 | na | Africa MA/ST | Late | CML454 | P27 | Lowland | Late |
| CML206 | REC | Africa MA/ST | Late | CML456 | REC | Highland | NA |
| CML207 | REC | Africa MA/ST | Late | CML458 | P87 | Highland | NA |
| CML208 | P92 | Africa MA/ST | Late | CML459 | REC | Highland | NA |
| CML210 | P43 | Africa MA/ST | Interm | CML461 | P88 | Highland | NA |
| CML211 | REC | Africa MA/ST | Late | CML463 | G9A | Transition | NA |
| CML212 | REC | Africa MA/ST | Early | CML464 | G9A | Transition | NA |
| CML214 | na | Africa MA/ST | Late | CML465 | AMATL | Asia Lowland | Late |
| CML215 | na | Africa MA/ST | Late | CML466 | P345 | Asia Lowland | Late |
| CML216 | na | Africa MA/ST | Late | CML468 | P28 | Asia Lowland | Late |
| CML217 | P31 | Africa Lowland | Early | CML471 | P147 | Asia Lowland | Interm |
| CML220 | P30 | Africa Lowland | Interm | CML472 | P147 | Asia Lowland | Interm |
| CML222 | P30 | Africa Lowland | Early | CML473 | P31 | Asia Lowland | Early |
| CML223 | SUWAN1 | Africa Lowland | Late | CML476 | P21 | Lowland | Late |
| CML224 | SUWAN1 | Africa Lowland | Late | CML477 | P43 | Lowland | Late |
| CML225 | SUWAN1 | Africa Lowland | Late | CML480 | SintAmTSR | Lowland | Interm |
| CML226 | SUWAN1 | Africa Lowland | Late | CML482 | G19 | Lowland | Interm |
| CML227 | SUWAN1 | Africa Lowland | Late | CML483 | P502 | Subtropical | Late |
| CML229 | SUWAN1 | Africa Lowland | Late | CML484 | MBRET | Subtropical | Late |
| CML235 | P44 | Africa Lowland | Late | CML490 | P63 | Lowland | Late |
| CML238 | P32 | Africa Lowland | Late | CML491 | REC | Lowland | Late |
| CML239 | P85 | Highland | Early | CML492 | P62 | Lowland | Interm |
| CML241 | P85 | Highland | Early | CML495 | RCW | Lowland | Late |
| CML243 | P85 | Highland | Early | CML499 | RCW | Lowland | Late/Interm |
| CML245 | P86 | Highland | Early | CML500 | RCW | Lowland | Late/Interm |
| CML247 | G24 | Lowland | Late | CML502 | RCWQ | Lowland | Late/Interm |
| CML252 | P21 | Lowland | NA | CML504 | Recycled | Africa MA/ST | Early/Interm |
| CML258 | P21 | Lowland | NA | CML508 | Recycled | Africa MA/ST | Early/Interm |
| CML259 | P21 | Lowland | NA | CML510 | Suwan | Africa MA/ST | Early/Interm |
| CML260 | P21 | Lowland | NA | CML512 | NA | Subtropical | Interm |
| CML261 | P21 | Lowland | NA | CML515 | NA | Lowland | Late |
| CML264 | P21 | Lowland | NA | CML516 | NA | Lowland | NA |
| CML268 | P23 | Lowland | NA | CML524 | P85 | Highland | Early |
| CML270 | P29 | Lowland | NA | CML525 | P85 | Highland | Early |
| CML271 | P29 | Lowland | NA | CML528 | NA | Highland | Early |
