# Supplementary material for: Genetic variation among elite inbred lines suggests potential to breed for BNI-capacity in maize
Source: Sci Rep. 2023 Aug 17;13:13422. doi: 10.1038/s41598-023-39720-3 (PMC10435450; doi:10.1038/s41598-023-39720-3)
Supplement: Supplementary file 11 — Supplementary Table 5. [file 41598_2023_39720_MOESM11_ESM.docx]

**Supplementary Table S5.** Minimum, mean and maximum diversity values (mrd average values) showed for the Collection (pop) and the Core Subset (core). Gain in mrd mean value expressed as a percentage of the collection mean value (Gain)

|  | **Core** | **Pop** | **Gain (%)** |
| --- | --- | --- | --- |
| **minimum** | 0.0301 | 0.0288 |  |
| **mean** | 0.1645 | 0.1637 | 0.7558 |
| **maximum** | 0.1845 | 0.1845 |  |
